# Exploring the influence of the microbiome on the pharmacology of anti-asthmatic drugs

**DOI:** 10.1007/s00210-023-02681-5

**Published:** 2023-08-31

**Authors:** Michael Chan, Chloe Ghadieh, Isphahan Irfan, Eamen Khair, Natasha Padilla, Sanshya Rebeiro, Annabel Sidgreaves, Vandana Patravale, John Disouza, Rachelle Catanzariti, Lisa Pont, Kylie Williams, Gabriele De Rubis, Samir Mehndiratta, Muralikrishnan Dhanasekaran, Kamal Dua

**Affiliations:** 1 Discipline of Pharmacy, Graduate School of Health, University of Technology Sydney, Ultimo, NSW 2007 Australia; 2 Department of Pharmaceutical Sciences and Technology, Institute of Chemical Technology, Matunga, Mumbai, Maharashtra India; 3 Department of Pharmaceutics, Tatyasaheb Kore College of Pharmacy, Warananagar, Tal: Panhala, Maharashtra 416113 India; 4 Present Address: Faculty of Health, Australian Research Centre in Complementary and Integrative Medicine, University of Technology Sydney, Sydney, Australia; 5 Drug Discovery and Development, Harrison School of Pharmacy, Auburn University, Alabama, USA

**Keywords:** Microbiome, Asthma, Corticosteroids, Montelukast, Azithromycin, Xenobiotics, Bioaccumulation, Biotransformation, Resistance, Pharmacotherapy

## Abstract

The microbiome is increasingly implicated in playing a role in physiology and pharmacology; in this review, we investigate the literature on the possibility of bacterial influence on the pharmacology of anti-asthmatic drugs, and the potential impact this has on asthmatic patients. Current knowledge in this area of research reveals an interaction between the gut and lung microbiome and the development of asthma. The influence of microbiome on the pharmacokinetics and pharmacodynamics of anti-asthmatic drugs is limited; however, understanding this interaction will assist in creating a more efficient treatment approach. This literature review highlighted that bioaccumulation and biotransformation in the presence of certain gut bacterial strains could affect drug metabolism in anti-asthmatic drugs. Furthermore, the bacterial richness in the lungs and the gut can influence drug efficacy and could also play a role in drug response. The implications of the above findings suggest that the microbiome is a contributing factor to an individuals’ pharmacological response to anti-asthmatic drugs. Hence, future directions for research should follow investigating how these processes affect asthmatic patients and consider the role of the microbiome on drug efficacy and modify treatment guidelines accordingly.

## Introduction

The human body’s microbiome comprises trillions of microorganisms, having an essential, unique role in maintaining health and homeostasis. The concept of microbiome linking with diseases is a growing branch of medical science, more specifically, how the microbiome effects physiology and pharmacokinetics (absorption, distribution, metabolism, and excretion of drugs) (Koppel et al. 2017). The link between microorganisms and pharmacokinetics is well documented, with articles published as far back as the 1970s (Goldman et al. 1974); and for instance, the phenomenon of digoxin inactivation by gut bacteria *Eubacterium lentum* is well documented (Saha et al. 1983).

Asthma is a chronic non-communicable condition characterized by variable respiratory symptoms due to airflow restriction caused by underlying inflammation or exposure to triggers (Papi et al. 2018). Symptoms of asthma include shortness of breath, chest tightness, coughing, and wheezing that can be induced by exercise and allergens; however, the root cause of asthma is yet to be discovered (Papi et al. 2018). Risk factors associated with asthma include epigenetic factors such as a family history of atopic disease (eczema, hay fever, and asthma) and exposure to tobacco smoke, airway microbes, and viruses (Papi et al. 2018). According to the World Health Organisation (WHO), asthma affected an estimated 262 million people in 2019 and was responsible for 455,000 deaths (World Health Organisation 2022). In Australia, asthma affected approximately 2.7 million Australians in 2020–2021 (Australian Bureau of Statistics 2020–21). More specifically, asthma is more prevalent in adult females than adult males, 12% to 9.4%, respectively, but is similar amongst both sexes in youth aged 0–14 (Australian Bureau of Statistics 2020–21). Worldwide, asthma affects over 300 million people and is more common in developed countries, including Australia, the United States, and the United Kingdom (Papi et al. 2018), with the highest prevalence report of 21.5%, belonging to Australia (To et al. 2012). However, the World Health Organization states that asthma-related mortality is more common in lower and lower-middle-income countries due to the lack of diagnosis and treatment (World Health Organisation 2022). In Australia, the indigenous population suffered from asthma 1.6 times more than the non-indigenous population (Versteegh et al. 2022).

Current treatment of asthma follows a step-wise approach based on symptom control and response to anti-asthmatic drugs (Papi et al. 2020). Medications involved in reducing airway inflammation and controlling patient symptoms include inhaled corticosteroids (ICS), long-acting beta-agonists (LABA), and short-acting beta agonists (SABA) for quick relief of asthma symptoms and emergencies (Papi et al. 2020). Then, 5–10% of asthmatic patients are unresponsive to inhaled and oral corticosteroids resulting in poor quality of life (Henderson et al. 2020). Moreover, we still do not understand why these subsets of people do not respond to corticosteroids (Henderson et al. 2020). Many theories have been suggested ranging from genetics to patient non-adherence (Jenkins 2019), but could the lung microbiome have a role to play (Barcik et al. 2020)? Barcik and colleagues in 2020 discussed the connection between the gut and lung and how the microbiota plays a bidirectional interaction between the two sites. The review alluded that the microbiome in the lung and gut impact asthma development and severity; however, the mechanism for achieving this was not identified. Another study done in 2018 by Sokolowska and associates suggested that the microbiome may influence the efficacy of specific therapeutics; however, how it affects inflammatory responses in asthmatic patients depends on the composition of the individual’s microbiome (Sokolowska et al. 2018).

Studies have shown the potential impact of corticosteroids on the gut and lung microbiome. In the lungs, *H. influenzae* is implicated in partially degrading inhaled corticosteroids and modifying host response to corticosteroids and can contribute to corticosteroid-resistant asthma (Durack et al. 2017; Goleva et al. 2013; Yang et al. 2018). To a lesser extent, in the gut, corticosteroid response has been shown to be influenced by microbes (Wang et al. 2021a, b). The lung microbiome also influences azithromycin; long-term azithromycin can help reduce asthma exacerbation frequency and severity (Thorsen et al. 2021), and studies have implicated a microbial link. Additionally, the degradation and bioaccumulation of montelukast by certain bacterial strains can impact the drug's metabolism.

While most research has focused on how microbiota metabolize pharmaceuticals as an undesirable effect, microbiota may also play a role in increasing the bioavailability and bioactivity of drugs. For instance, a study on plant based medicines, low-bioavailable ginsenosides were biotransformed by intestinal microbiota to form a metabolite, Compound K, which showed increased bioactivity when compared to the parent compound (Wang et al. 2011) (Wang et al. 2011). Compound K is an active metabolite of protopanaxadiol ginsenoside, a molecule present in ginseng extract, which is formed by intestinal microflora following oral administration of the extract and reaches systemic circulation (Choi et al. 2016). Therefore drug–microbiome interactions should be considered in future studies, as it could have potentially beneficial effects which may vary among individuals.

Corticosteroids both inhaled and oral, montelukast, and azithromycin as anti-asthmatic drugs are focused on in this review due to the availability of literature surrounding this niche topic. However other anti-asthmatic drugs are used such as short and long acting beta_2_-adrenergic agonists (SABA and LABAs), short- and long-acting muscarinic antagonists (SAMA and LAMAs), and biologic treatments are also used in the treatment of asthma but we were unable to find sufficient literature on the influence of the microbiome on these drugs (Global Initiative for Asthma 2022).

Current research indicates significant potential for microbiome–drug interactions, especially in the gut; however, research is lacking in the areas of how the microbiome interacts in the lungs and how microbes interact with anti-asthmatic drugs. More research is needed to investigate how the microbiome and asthma interplay as current research suggests that the microbiome has the capacity to alter patient treatment outcomes such as modulating response to and altering the pharmacokinetics of anti-asthmatic drugs. Further research will especially benefit people with more targeted treatments, especially patients with corticosteroid-resistant, severe, and difficult-to-treat asthma, where current treatment options are unable to sufficiently control symptoms and exacerbations leading to hospitalizations and worse quality of life.

Clearly, the microbiome is increasingly intertwined with pharmacology; however, current literature on asthma treatment neglects the impact of the microbiome on treatment pharmacology. So how does this affect the therapeutic approach for asthmatic patients? In this literature review, we attempt to discuss how the microbiome influences the pharmacology of anti-asthmatic drugs, particularly oral and inhaled corticosteroids, montelukast, and azithromycin. Moreover, we delve into how biotransformation and bioaccumulation impact drug metabolism, resistance, and efficacy. We also aim to provide an overview of the existing literature on how the microbiome affects the pharmacokinetic, pharmacodynamic, and pharmacogenomic processes.

## Role of the microbiome

Microbiota play a vital role in both health and disease. The symbiotic relationship between the human body and microbial community is quite important for proper immune function and health (Chellappan et al. 2019). These microbiomes assist in the digestion of food, regulation of the immune system, protection against disease-causing bacteria, and production of amino acids and vitamins. Every surface of the body has a microbiome keeping balance, and it is now known that these communities can interact (Mohajeri et al. 2018).

### Gut–lung axis

The concept of gut–lung axis refers to the complex bidirectional communication that occurs between the microbiota of the gut and lungs. Current advances in the exploration of human microbiota have led to an increase in the understanding of the various communities of microbes and the ways in which they interact (Enaud et al. 2020). The axis allows for the movement of hormones, endotoxins, cytokines, and microbial metabolites into the bloodstream between both microbiomes. According to Zhang and co-writers, studies have shown that the gut microbiota can affect pulmonary immunity through the cross-talk occurring between the gut and lung microbiota (Zhang et al. 2020). Similarly, inflammation occurring in the lungs can trigger a change in the blood and gut microbiota. The crucial role of lung microbiota has only been researched over the last few years. The mechanisms and extent to which the microbiota influences the pathogenesis of lung diseases are still being extensively studied (Shukla et al. 2019).

### Drug transformation

**Fig. 1:**
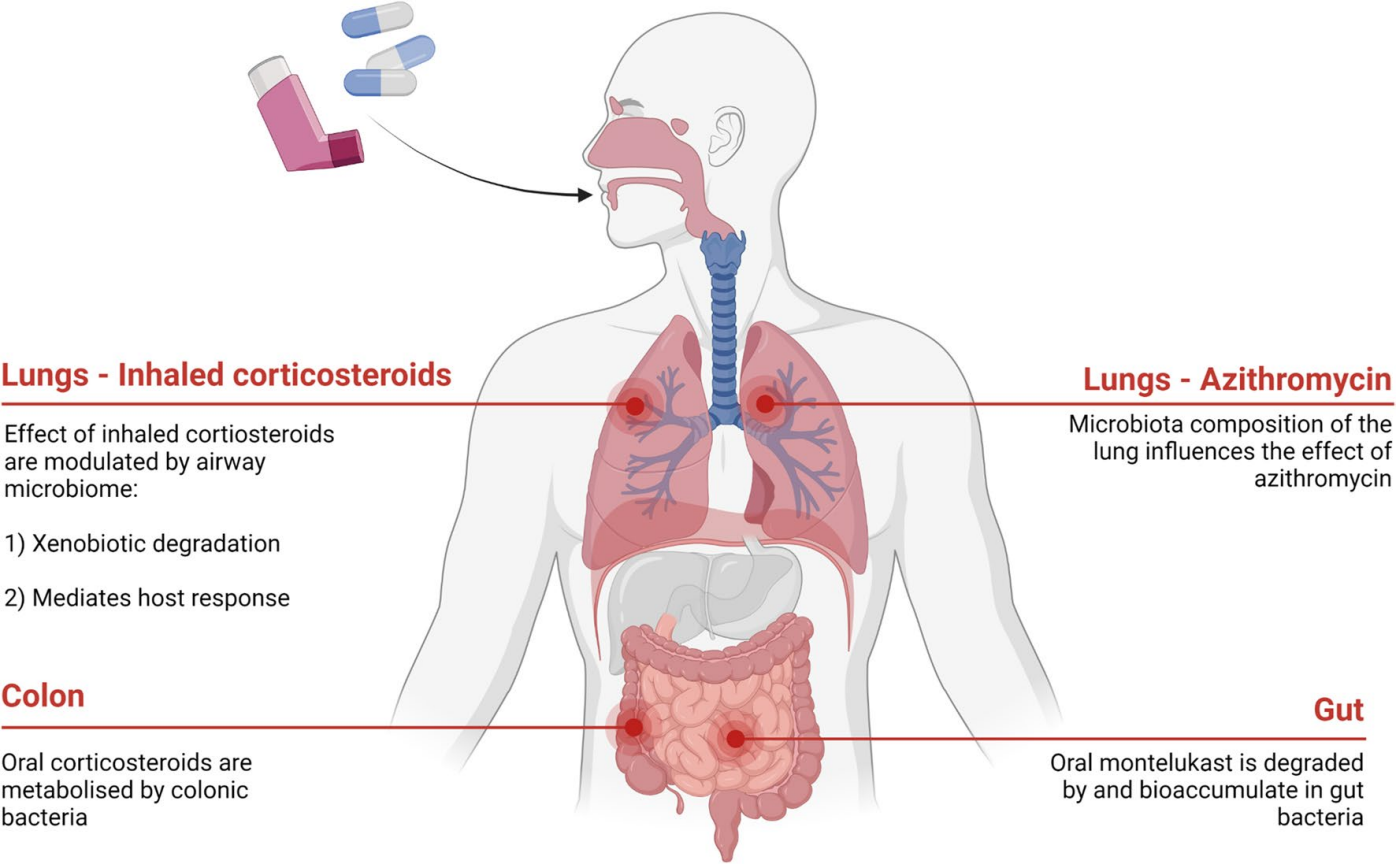
Influence of lung, gut, and colon microbiota on anti-asthmatic drugs. Drugs used to treat asthma are influenced by the microbiome in various physiological systems such as the lungs, colon and gut which influences the pharmacology of these drugs

In addition to the role of microbiota in disease progression, recent studies have identified its influence in transforming pharmaceuticals (Fig. 1). The human microbiome is inextricably linked to altered pharmacokinetic properties of drugs (Koppel et al. 2017). The microbial inhabitants of the microbiota possess metabolic properties which convert drugs into active, inactive, or toxic metabolites (Hitchings & Kelly 2019). This process subsequently influences parameters like bioavailability and leads to various clinical outcomes (Cussotto et al. 2021). Majority of current research exploring this phenomenon is focused on the human gut microbiome (HGM); the largest constituent of the overall human microbiome (McCoubrey et al. 2022). Initial studies established the role of the microbiota in modifying the efficacy of certain drugs. One such study identified Levodopa, a treatment involved in Parkinson’s disease, as undergoing microbial transformation (Matthewman et al. 2022). Two bacteria isolated from the gut, *Enterococcus faecalis* and *Eggerthela lenta*, were found to metabolize Levodopa before it crossed the blood–brain barrier, ultimately rendering it ineffective (Maini Rekdal et al. 2019). An additional study conducted by Zimmermann et al. expanded on this concept, analyzing the role of 76 different gut microbes and their ability to metabolize 271 drugs. The in vitro study involved grouping the 271 drugs into 21 drug pools, each incubated with the 76 gut microbial strains for 12 h under anaerobic conditions. Drug concentrations were measured using liquid-chromatography-coupled mass spectrometry (LC–MS) before and after incubation (Zimmermann et al. 2019). Of the 271 drugs, 176 were metabolized so significantly that the level of these drugs decreased by more than 20% over the indicated timeframe (Zimmermann et al. 2019).

More recently however studies have begun to target specific drugs, and the relevant microbes which contribute to their modification. For example, Cussotto et al. examined the role of gut microbiota in determining the bioavailability of olanzapine, with initial results exhibiting a direct correlation (Cussotto et al. 2021). In-depth analysis of this reaction revealed *Alistipes* as the relevant genus associated with olanzapine modification. Moreover, additional studies have expanded this area of research by establishing causal links between certain pharmaceuticals and distinct microorganisms of the gut microbiota. Such studies provide the basis for more comprehensive research, whereby the direct mechanisms which underpin the influence of said organisms on certain drugs can be identified. As such, Wang et al. analyzed the importance of gut microbiota in facilitating varied interpatient response to statin treatment. A decrease in *Akkermansia muciniphila* and *Lactobacillus* accompanied by a simultaneous increase in *Holdemanella* and *Facecallibacterium* was linked to poor outcomes in statin treatment. However, a direct understanding of the transformation of statin pharmacokinetic properties is limited and thus warrants further research (Wang et al. 2021a, b).

Overall, the research highlights the importance of the human gut microbiome as an area of study when considering the influence of microbiota on drug transformation. However, by focusing primarily on the gut microbiota other important niches in the microbiome, such as the lung microbiota, have remained under-researched. Therefore, the remainder of this review aims to not only explore the current research available on the lung and gut microbiota, with a direct focus on asthma treatment, but the limitations of such research and potential areas for future study.

## Corticosteroids

Corticosteroids are synthetic analogues of the natural steroids, glucocorticoids and mineralocorticoids, and are used as first line anti-inflammatories for many inflammatory diseases (Barnes 2006). Corticosteroids possess and display properties from both these steroids to mediate an anti-inflammatory effect. Glucocorticoids have immunosuppressive, anti-inflammatory and vasoconstrictive effects (Timmermans et al. 2019), and mineralocorticoids control the balance between water and electrolytes during renal filtration (Belden et al. 2017). These anti-inflammatory properties make corticosteroids mainstream therapy for asthma which is primarily characterized by bronchial inflammation (Alangari 2014).

Due to the categorisation of asthma as an inflammatory disease, corticosteroids are an effective therapy as they can be administered orally or inhaled. Oral corticosteroids are given early in the treatment of an acute asthma exacerbation and have shown to be the most effective for fast symptomatic relief (Alangari 2014). In comparison, ICS are used as maintenance therapy for patients with recurring symptoms (Volmer et al. 2018). Oral corticosteroid use has been linked with side effects such as increased risk of infection and osteoporosis. As such, when administering oral corticosteroids, clinicians should aim toward short-term use at lower doses until the therapeutic goal is reached (Volmer et al. 2018).

### Structure activity relationship and mechanism of action

Corticosteroids work to relieve bronchial muscle spasms by binding to the glucocorticoid receptor which affects multiple inflammatory pathways and the regulation of the β_2_-adrenergic receptor (Townley & Suliaman 1987). This receptor is predominantly located intracellularly in the cytoplasm, which upon binding with the corticosteroid, migrates into the nuclear compartment. It inhibits gene expression, transcription, and translation of inflammatory leukocytes and epithelium, limiting the production of inflammatory cytokines and chemokines which hinders the inflammation process. Thereby inhibiting mucosal oedema and bronchial smooth muscle contraction and thus reducing bronchoconstriction and airway reactivity (Bucca & Rolla 1989; Williams 2018).

### Inhaled corticosteroid pharmacokinetics

After inhalation only about 40 to 60% of the ICS administered reaches the lungs (dependent upon inhalation technique). The rest is swallowed by the patient and absorbed through the gastrointestinal tract (Padden et al. 2008). Corticosteroids are then distributed within the lungs to areas that have a high density of glucocorticoid receptors such as alveolar walls endothelium and the smooth muscles of pulmonary and bronchial vessels (Lipworth 2000). The amount of ICS that is swallowed is metabolized in the liver via first pass metabolism and a small portion of drug is then released into systemic circulation (Barnes 2010; Padden et al. 2008). The portion of drug that was delivered straight to the lungs does not need to go through first pass metabolism via the liver and is metabolized in the lungs for local effect (Moore et al. 2013). ICS when swallowed are heavily metabolized into inactive metabolites which are then excreted in urine.

### Influence of microbiome on inhaled corticosteroids

Goleva et al. first reported in 2013 that certain bacteria elevated in asthmatic patients were associated with reduced corticosteroid response and was only found in corticosteroid-resistant asthmatics (Goleva et al. 2013). Differences at genus level were found between corticosteroid-resistant and corticosteroid-sensitive asthmatics. The study highlighted interactions between *Haemophilus parainfluenzae* and bronchoalveolar lavage macrophages. When the bronchoalveolar lavage macrophages were cultured together in the presence of *H. parainfluenzae*, there was an increase in levels of p38 mitogen-activated protein kinase activation, a measure of inflammation (Pelaia et al. 2021), and reduced cellular responses to corticosteroids (Goleva et al. 2013). Previous studies found a relationship between *H. influenzae* and steroid-resistant neutrophilic asthma (Essilfie et al. 2012, 2011), However, the functional interactions between host and microbiome were not investigated. Consequently, Goleva et al. showed that microbial composition affected the patient’s response to corticosteroids by influencing the responses of airway macrophages.

Furthermore, Yang et al. in a murine model found that long-term *H. influenzae* exposure during allergic airway disease caused multifaceted changes to the airway. Importantly, exposure converted the steroid sensitive airway inflammation phenotype to one that is steroid-resistant. Moreover, exposure created an altered inflammatory response through inhibiting regulatory T cell associated immunosuppression without affecting Th1-associated inflammation in addition to affecting phagocytosis of airway macrophages which the authors hypothesized could contribute to excessive neutrophilic inflammation (Yang et al. 2018). This study found that long-term exposure to *H. influenzae* may play a role in asthma pathogenesis and ICS resistant airway inflammation. In the context of this review, this study further adds to the evidence that *H. influenzae* is involved in the pathophysiology of asthma and that the microbial composition of patients could affect their sensitivity to ICS.

Durack et al. studied patients who had never used ICS before and measured their bronchial microbiome, followed by their response to ICS and then analyzed their bronchial microbiome after ICS therapy. The treatment included 6 weeks of either twice daily inhaled fluticasone propionate 250 mcg or placebo. Compositional differences in patients with atopic asthma, non-atopic asthma, and healthy individuals were reported. Of note, there were no differences between the groups in other microbial metrics such as richness, evenness, and microbial burden, which further supports that differential abundances and microbiome related functions are related to ICS responsiveness rather than non-specific numbers of microbes or broad diversity measurements (Durack et al. 2017).

**Fig. 2:**
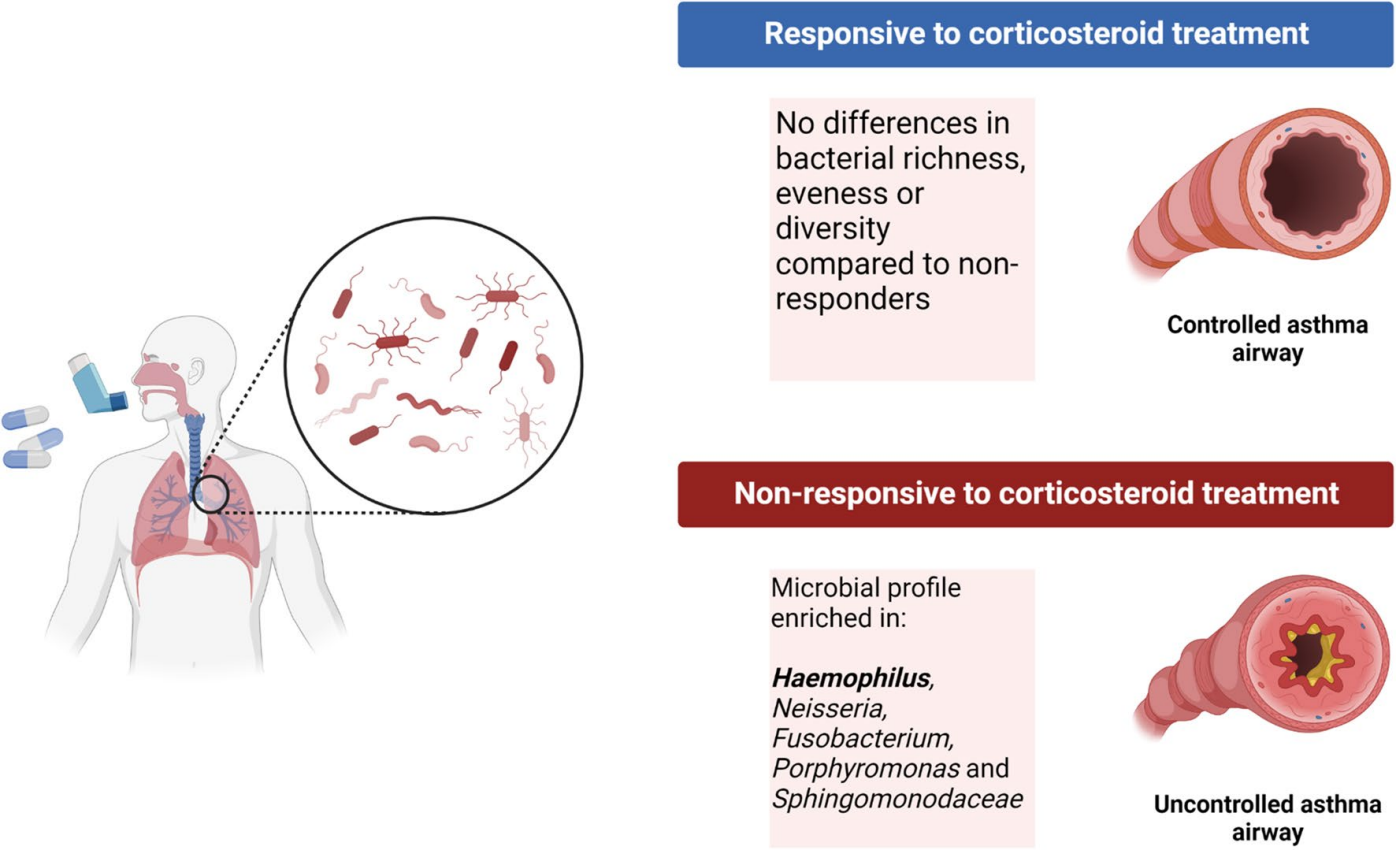
Corticosteroid treatment responsiveness can be predicted by bacterial differential abundance. Bacterial differential abundance is associated with ICS treatment responsiveness, patients with corticosteroid-resistant asthma are associated with a microbial profile enriched in *Haemophilus, Neisseria, Fusobacterium, Porphyromonas, and Sphingomonodaceae,* whereas there was no association between bacterial richness, evenness of diversity in ICS responsiveness

The analysis revealed that asthmatics are more likely to have enrichment in the following microbes: *Haemophilus, Neisseria, Fusobacterium, Porphyromonas* and *Sphingomonodaceae,* and depleted in members of the *Mogibacteriaceae* and *Lactobacillales *(Fig. 2). The study also looked at the predicted functional profiles of these enriched microbes and found that these bacteria had enriched predicted functions in the metabolism of short-chain fatty acids, which are associated with anti-inflammatory properties in the gut. They also found that some of these microbes were enriched in predicted functions involved in xenobiotic degradation. Some of those enriched species were already shown to reduce responsiveness to corticosteroids in previous studies, namely *Haemophilus*.

Together, this shows that asthmatic patients have microbial profiles that are different to healthy non-asthmatics. They also showed that patients who do not respond to inhaled corticosteroids are enriched in certain bacteria. Furthermore, the predicted functions of these bacteria involve processes to both degrade the corticosteroids and interfere with the patient’s physiological responses.

Thus, Durack et al. have shown that firstly, the pharmacokinetic process of metabolism can occur in the lung microbiome, which can potentially affect the absorption of ICS, consequently decreasing the effect of ICS. Secondly, they have demonstrated that asthmatic patients are enriched in microbes associated with reducing the host response to corticosteroids, though the exact causal mechanism is not known.

Another consideration is whether the microbiome has a role in modulating adverse effects for instance, in the increased risk of pneumonia during ICS use. Numerous studies have found that ICS use increased the risk of developing pneumonia in chronic obstructive pulmonary disease (COPD) patients (Chen et al. 2021; Leitao Filho et al. 2021). As shown by Durack et al., the microbiome could potentially affect the efficacy and response of ICS, potentially modifying the immunosuppressive and anti-inflammatory processes which may contribute to pneumonia risk. Whether the microbiome plays a role in the risk of developing pneumonia because of changes to pharmacology is to be determined.

### Influence of microbiome on oral corticosteroids

**Fig. 3:**
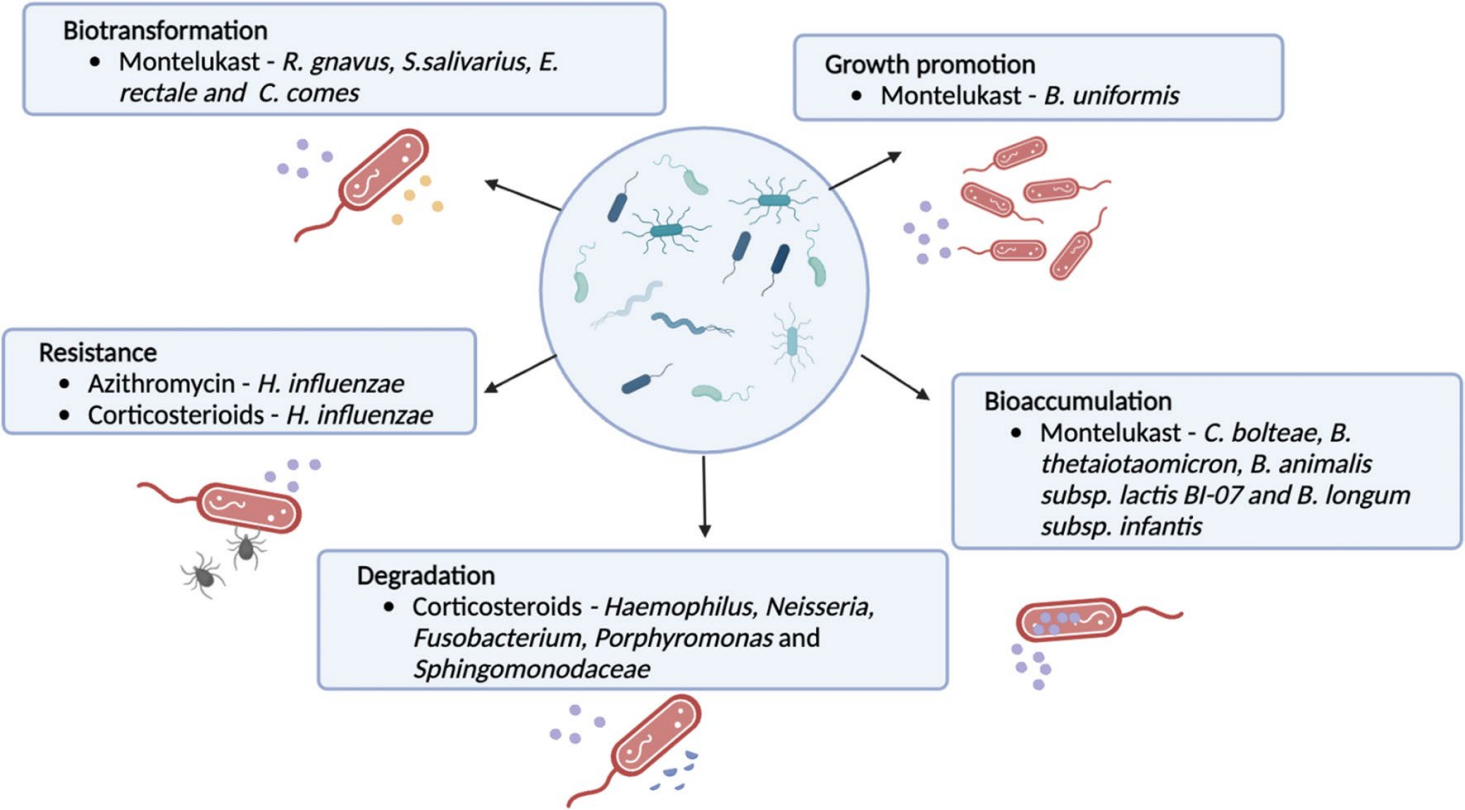
Bacterial interactions with corticosteroids, azithromycin, and montelukast. Bacteria are involved in various processes that affect the pharmacology of drugs. Gut bacteria are capable of biotransforming and bioaccumulating montelukast while montelukast can promote the growth of certain bacteria. Secondly, bacteria have been shown to degrade corticosteroids in the gut. Thirdly, the microbial profile of the lungs is associated with responsiveness to treatment of azithromycin and ICS

Even though oral corticosteroids are rapidly absorbed and do not generally get absorbed in the colon, oral corticosteroids such as prednisolone are capable of being degraded by the microbiome (Yadav et al. 2013). Moreover, other corticosteroids used in ICS pharmacotherapy, such as budesonide, are biotransformed by colonic microbiota; this adds to the evidence of the potential for microbes in physiological systems to have the capacity to biotransform corticosteroids (Figs. 2 and 3).

Additionally, interactions between the gut microbiome and efficacy of corticosteroids have been documented in an animal model of lupus. In one study by Wang and colleagues, mice were treated with three doses of prednisone to determine the effect on the gut microbiota; then, a fecal microbiota transplant (FMT) was performed to transfer the gut microbiota of the prednisone mice to untreated mice. They found that FMT-treated mice had improvements to their lupus, despite not being treated with prednisone, and did not show the side effects of prednisone treatment. Thus, they found that prednisone-regulated gut microbiota could play a role in the therapeutic effects of prednisone (Wang et al. 2021a, b). However, this study was based on an animal model and was not specific to asthma, nor did the study determine the causal mechanisms behind the alleviation in lupus. Nonetheless, this study showed that the gut microbiome has the potential to attenuate prednisone response in patients, whether these findings are applicable to human asthmatic patients is to be determined.

## Montelukast

Montelukast can be used as an adjunct to ICS therapy or as an alternate prophylactic treatment for asthmatics (Zuberi et al. 2020). It improves long-term asthma management when combined with ICS (Zuberi et al. 2020) and appears to be very effective in treating some phenotypes of asthma, such as exercise-induced asthma and asthma linked with rhinitis (Philip et al. 2004). Although less effective than regular low-dose ICS therapy, montelukast can be used as monotherapy for individuals who either have experienced adverse reactions to ICS, patients with concurrent allergic rhinitis or for whom inhaled therapy is impractical (Joos et al. 2008). Dosing for adults and adolescents is 10 mg in tablet form taken orally once daily (Green 2006; Jarvis & Markham 2000). Although no significant drug interactions have been identified, individuals with mental health conditions (e.g., depression) should be managed with extra caution since the medication has the potential to alter behaviour and mood (Fanta 2022).

### Structure activity relationship and mechanism of action

Leukotrienes are one of the several inflammatory molecules released by mast cells during an asthma attack and are primarily responsible for bronchoconstriction (Berger 1999). Leukotriene receptor antagonists exert their biological effect by binding with high affinity and selectivity to cysteinyl leukotriene receptor type 1 (CysLTR1), a G protein coupled to CysLTR2, on the surface of bronchial smooth muscle cells (Tavares et al. 2021). As a result, it inhibits any physiological effects of CysLTs such as LTC4, LTD4, and LTE4 at the receptor that can trigger asthma (Green 2006). Montelukast is a leukotriene receptor antagonist that functions by obstructing leukotriene D4 in the lungs, therefore, reducing inflammation and relaxing smooth muscles to widen airways (Benninger & Waters 2009). Clinical trials have revealed that montelukast protects against bronchoconstriction by improving asthma symptoms, preventing exacerbations, and reducing eosinophil levels in the blood (Paggiaro & Bacci 2011).

### Montelukast pharmacokinetics

Montelukast, following oral administration, is characterized by an immediate, almost exhaustive absorption phase. It possesses an average oral bioavailability of approximately 64% and takes 3 h to reach the mean peak plasma concentration in a 10-mg tablet. Moreover, the distribution of montelukast is significantly associated with its ability to bind to plasma proteins, i.e., montelukast is bound to more than 99% of plasma proteins. However, montelukast’s metabolism occurs extensively in the liver and is predominantly mediated by the Cytochrome P450 2C8. Subsequently, excretion of montelukast, and its metabolites, occurs (almost entirely) via the bile (MIMS Australia 2022).

### Influence of the microbiome on montelukast

Klünemann et al. reported that montelukast was bioaccumulated by some bacteria such as *Clostridium bolteae* and biotransformed by others (Table 1). They also observed that montelukast promoted the growth of the bacteroides *B. uniformis* (Klünemann et al. 2021)*.* The study examined how interactions between 25 different strains of human gut bacteria and 15 different drugs affected drug availability and metabolism. Bioaccumulation refers to the process where a bacterium retains a compound intracellularly without chemically altering it and has a reduced impact on bacterial proliferation (Cohen & Kelly 2022), whereas biotransformation chemically modifies the compounds causing toxicity and affecting drug availability (Cohen & Kelly 2022). Furthemore, xenobiotic bioaccumulation of *Clostridium bolteae* and the growth promotion of *B. uniformis* was also reported in a similar study (Lindell et al. 2022). Xenobiotics directly impact the gut microbiota's composition and metabolic activity as gut bacteria can metabolize xenobiotics or bioaccumulate, which alters their activity or toxicity (Lindell et al. 2022). How xenobiotic metabolites are influenced by gut microbiota depends on their composition (Lindell et al. 2022). Hence, these interactions could explain other findings of drug depletion without the presence of a drug metabolism product (Lindell et al. 2022). However, both studies failed to explain how these interactions affected the metabolism of montelukast when used for asthma. Thus, further research is required to understand the role of xenobiotics and gut microbiota in the bioaccumulation of montelukast.

**Table 1:** Summary of studies where the microbiome affects drug response

Location	Mechanism	Key findings	Reference
Lung	Microbes reduce inhaled medication pharmacodynamics by influencing cellular response	*Haemophilus* genus bacteria enrichment reduced patient response to inhaled fluticasone by influencing monocyte/macrophage activation which reduced cellular response to corticosteroids	(Goleva et al. 2013)
		Long-term exposure to *Haemophilus influenzae* is associated with steroid-resistant neutrophilic asthma in a mice model	(Yang et al. 2018)
Lung	Microbes reduce medication response to ICS by bacterial metabolism of xenobiotics which inactivates drug	Certain bacteria increase xenobiotic degradation, diminishing the response of ICS, possibly by degradation of the drug. Patients non-responsive to ICS have bacterial communities enriched with xenobiotic degradation pathways	(Durack et al. 2017)
Lung	Microbiota composition of the lung influences the effect of azithromycin	Microbiome influenced duration and effect of azithromycin in asthma-like symptoms in children	(Thorsen et al. 2021)
Gut	Montelukast is degraded by and bioaccumulate in gut bacteria	Bioaccumulation of montelukast can attenuate the host drug response Gut bacteria identified as being capable of biotransforming montelukast	(Klünemann et al. 2021)
		Montelukast causes growth promotion	(Lindell et al. 2022)
Gut	Oral corticosteroids are metabolized by colonic bacteria	In vitro study showed that simulated colonic bacteria can completely degrade prednisolone in 3 h	(Yadav et al. 2013)
Gut	Prednisone efficacy and side effects are mediated by gut microbiota	In a lupus animal model, the microbiome can influence the therapeutic efficacy and side effects of prednisone	(Wang et al. 2021a, b)

## Azithromycin

Azithromycin is a macrolide antibiotic with antibacterial, antiviral, and anti-inflammatory effects that is used in the treatment of asthma (Gibson et al. 2017). Macrolides have multiple immunomodulatory effects that are used to treat many chronic inflammatory diseases. For example, asthma exacerbations can be caused by chronic neutrophilic inflammation while macrolides are understood to reduce neutrophilic inflammation; therefore, it is reported that azithromycin’s immunomodulatory effects are utilized to treat asthma (Zimmermann et al. 2018). Dosage of azithromycin for asthma exacerbation control have been studied at 500 mg (Gibson et al. 2017) or 250 mg (Brusselle et al. 2013) three times a week, taken orally as tablets (Global Initiative for Asthma 2022). Low-dose azithromycin is used in adults as an add-on treatment for the management of severe asthma (Global Initiative for Asthma 2022), and azithromycin is used long-term in patients with persistent symptomatic asthma despite standard treatment of ICS/LABA to reduce asthma exacerbations and improve quality of life (Gibson et al. 2017; Hiles et al. 2019). Azithromycin, being an antibiotic, obviously has significant interplay with the microbiome, for instance, long-term use of azithromycin reduces airway proportion of *H. influenzae* and increased antibiotic resistance to macrolide antibiotics (Taylor et al. 2019).

### Structure activity relationship and mechanism of action

Azithromycin works in two ways, firstly by preventing peptide elongation and secondly, targeting 50S ribosomal subunit assembly thereby inhibiting bacterial protein synthesis (Mabe et al. 2004; Parnham et al. 2014). Macrolides form hydrogen bonds between the 23S rRNA and groups on the desosamine sugar and the lactone ring, and azithromycin is further capable of forming hydrogen bonds with the azalide group (Mabe et al. 2004).

### Azithromycin pharmacokinetics

Azithromycin has approximately 37% gastrointestinal absolute bioavailability. Azithromycin is widely distributed in the body; moreover, azithromycin has a high affinity for the tonsil, lung, prostate, liver, and lymph node tissue and tends to concentrate in macrophages and polymorphonuclear leukocytes. Metabolism is hepatic demethylation and urinary excretion unchanged drug accounts for less than 6% of excretion; excretion is primarily biliary (Drew & Gallis 1992; Lode 1991).

### Influence of the microbiome on azithromycin

Thorsen et al. reported in 2021 that azithromycin effectiveness was mediated by airway microbiota richness. They found that in preschool children with asthma-like symptoms, the airway microbiota affected the effectiveness of azithromycin in reducing asthma-like symptoms (Table 1). High microbiota richness was associated with increased effectiveness of azithromycin (Thorsen et al. 2021), suggesting some patients may benefit more from azithromycin treatment than others. They measured microbial richness by number of operational taxonomic units and found that there was a 10% increased effect of azithromycin for every 10 additions OTU’s, with effect measured by participants with continued asthma-like symptoms.

Azithromycin efficacy is affected by the airway microbiome (Figs. 1 and 3). When used in the treatment of asthma, azithromycin is not reliant on just the anti-inflammatory effects; its antibacterial effects play a significant role alongside the anti-inflammatory and antiviral effects.

Similarly, a study by Combs et al. found that in lung transplant patients, lung bacterial burden and microbial composition predicted patient response to treatment and azithromycin (Combs et al. 2021). Together, this reinforces the impact of the lung microbiome as a key factor for azithromycin pharmacotherapy in lung diseases, and that the effectiveness of azithromycin for treating asthma is modulated by the patient microbiome. However, the pharmacological mechanistic cause is unknown, and research has suggested that the richness of the lung microbiome is key to responsiveness to treatment. Table 1 summarizes the key findings of the effect of microbiota, its location, and effect on various treatment drugs like corticosteroids, antibiotics and montelukast.

## Strengths and challenges

In this review, we have considered how the microbiome can potentially affect the function of drugs. However, research in this area is limited. Conversely, the reverse topic of how drugs affect the microbiome is an area of vigorous research (Hartmann et al. 2021). Research on the lung microbiome is another limiting factor, as it was traditionally believed that the lungs were sterile. However, through the development of metagenomics, we now know the lungs are host to various microorganisms, from bacteria to fungi and viruses (Yagi et al. 2021).

Moreover, in this review, we only considered the bacterial microbiome because existing literature focuses on the bacterial microbiome. The fungal or viral microbiome is an under-researched area, despite being increasingly implicated in disease pathophysiology (Mukhopadhya et al. 2019; Pérez 2021). Another challenge is the lack of mechanistic causal studies directly linking the microbiome to pharmacological influences impacting patients.

Furthermore, the findings from Klünemann et al. and Lindell et al. demonstrate that montelukast influences bioaccumulation and growth promotion of specific bacterial strains (Table 1); however, it is limited by the small sample size utilized. Moreover, the study by Klünemann et al. mainly focused on duloxetine, which could have a different mechanism of bioaccumulation by gut bacteria compared to other drugs. Overall, the findings imply the need for a more comprehensive mapping of drug interactions with human gut microbiota, particularly anti-asthmatic drugs.

## Conclusion and future perspectives

We have discussed the multifaceted ways that the microbiome affects anti-asthmatic drugs; however, our understanding of the clinical implications of these findings is limited. Existing literature highlights how the gut and lung microbiome impacts asthma development and severity; however, the impact of lung microbiome on the pharmacology of drugs shows research gaps.

Pharmacotherapy is crucial in the treatment of asthma; however, some patients are less sensitive to treatment and the mechanistic causal reason is yet to be completely determined; though it is thought to be a combination of factors such as genetics and inhaler technique, a significant proportion of asthmatics are resistant to corticosteroids, and in our review, we have probed into how the microbiome could potentially play a role in corticosteroid resistance, though the microbiome is only one part of the bigger picture. Moreover, the bacterial richness of the lungs can play a significant role in the efficacy of azithromycin. Future research should investigate mechanistically how these processes affect asthmatic patients and incorporate the microbiome as a consideration when investigating anti-asthmatic drugs.

Furthermore, bioaccumulation and biotransformation due to the presence of specific gut bacteria strains can potentially alter metabolism and thus influence drug efficacy. However, the mechanism of this interaction with montelukast is yet to be explained. Large-scale in vitro studies can achieve this to understand the association between gut and lung microbiome and how it impacts the efficacy of anti-asthmatic drugs. Emerging studies need to consider confounding factors such as lifestyle, diet, medical conditions and other regular medicines. Moreover, further research should identify firstly on the mechanistic pathways in which the microbiome specifically affects the pharmacology in patients, and secondly, how we can utilize these pharmacological processes to translate into more effective treatment options for patients. Future studies should incorporate the lung microbiome as a consideration, especially in the field of ICS.

Figures 1 and 3 were created with BioRender.com. Figure 1 was adapted from “Health Effects of Air Pollution”, by BioRender.com (2022), retrieved from https://app.biorender.com/biorender-templates. Figure 2 was adapted from “Coronary Artery Disease”, by BioRender.com (2022), retrieved from https://app.biorender.com/biorender-templates.

## Data Availability

This is not applicable.

## Abbreviations

ICS: Inhaled corticosteroids
SABA: Short-acting beta agonist
LABA: Long-acting beta agonist
ICS/LABA: Inhaled corticosteroids and long-acting beta agonist combination treatment
SAMA: Short-acting muscarinic antagonist
LAMA: Long-acting muscarinic antagonist
OTU: Operational taxonomic unit
WHO: World Health Organization
FMT: Fecal microbiota transplantation
CysLTR1: Cysteinyl leukotriene receptor type 1
